# Effects of isolated or combined carbohydrate and caffeine supplementation on tennis training performance: single-blind randomized placebo-controlled crossover session

**DOI:** 10.3389/fnut.2025.1608893

**Published:** 2025-07-14

**Authors:** Mekki Abdioglu, Ahmet Mor, Dan Iulian Alexe, Raul Marian Todor, Elena Adelina Panaet, Cristina Ioana Alexe, Firat Akca

**Affiliations:** ^1^Graduate School of Health Sciences, Ankara University, Ankara, Türkiye; ^2^Department of Coaching Education, Faculty of Sport Sciences, Sinop University, Sinop, Türkiye; ^3^Department of Physical and Occupational Therapy, “Vasile Alecsandri” University of Bacau, Bacău, Romania; ^4^Department of Environmental Science, Physics, Physical Education and Sport, ”Lucian Blaga” University of Sibiu, Sibiu, Romania; ^5^Department of Physical Education and Sports Performance, “Vasile Alecsandri” University of Bacău, Bacău, Romania; ^6^Department of Coaching Education, Faculty of Sport Sciences, Ankara University, Ankara, Türkiye

**Keywords:** groundstrokes, ergogenic aids, tennis, performance, training

## Abstract

**Background:**

In long tennis matches, the number of unforced errors in groundstrokes increases. However, players need to maintain their successful strokes consistently in order to be successful in matches. To overcome this situation, tennis players utilize certain ergogenic supplements. In order to determine the most effective ergogenic supplement on players’ performance, it is aimed to investigate the effects of caffeinated chewing gum (CAF_GUM_), carbohydrate gel (CHO_GEL_) and cho gel + cafe gum (CHO_GEL_ + CAF_GUM_) on tennis players’ basic strokes, countermovement jumps (CMJ), heart rate (HR), ratings of perceived exertion (RPE) and gastrointestinal discomfort in a training session.

**Method:**

The study included 14 male tennis players (mean age: 15.93 ± 0.83 years, height: 173.86 ± 6.89 cm, and body mass: 60.64 ± 2.58 kg) with experience in national and international tournaments. Players ingested CHO_GEL_ (21.1 g) + CAF_GUM_ (100 mg) or CHO_GEL_ (21.1 g) + gum placebo (PLA_GUM_) or CAF_GUM_ (100 mg) or PLA_GUM_ before a high-intensity workout and at the end of each set.

**Results:**

The CHO_GEL_ + CAF_GUM_ session performed better groundstrokes than the control (CON) (*p* = 0.001) and the PLA_GUM_ sessions (*p* = 0.001). When total RPE values were considered in the training session, the CHO_GEL_ + CAF_GUM_ session had lower RPE scores than the CON (*p* = 0.010) and PLA_GUM_ (*p* = 0.044) sessions. The CHO_GEL_ + PLA_GUM_ session had significantly lower RPE scores than the CON (*p* = 0.005) and PLA_GUM_ (*p* = 0.005) sessions. The CAF_GUM_ session had significantly lower RPE scores than the CON (*p* = 0.013). It was observed that no supplements significantly affected either HR (*p* = 0.188) or CMJ (*p* = 0.349) scores.

**Conclusions:**

In conclusion, there was a significant difference on basic strokes and RPE scores between CHO_GEL_ + CAF_GUM_ supplementation used before and during training compared with the control session. At the same time, there was no significant performance outcomes between CHO_GEL_ and CAF_GUM_ sessions.

## 1 Introduction

In tennis matches or training, players quickly perform high intensity running and explosive groundstrokes. A tennis match usually lasts 1–1.5 h, but some matches can last up to 5 h (1). In matches lasting more than 1.5 h or long-term training sessions, players may experience increased internal load (heartbeat, lactic acid etc.) due to repeated high-intensity runs, acceleration, and deceleration (2, 3). This situation may disrupt the homeostatic balance in players, and the player’s performance may be negatively affected. In some studies, on this issue, fatigue caused by high-intensity activities reduced tennis players’ groundstrokes by up to 69% (4). In another study, players’ groundstrokes decreased by up to 81% in a 92-min match (5). Horney et al. (3) showed that players’ nutritional status before and during intense high-intensity training or prolonged matches significantly impacts their current performance. In another study, 63.9% of tennis players reported experiencing hypoglycaemic symptoms during a tennis tournament or tennis training (6). Tennis players use ergogenic aids such as carbohydrate (CHO) or caffeine (CAF) to maintain and improve their performance during long, hard training sessions or matches (7). Caffeine use is increasing, especially among tennis players in the top 100 worldwide (7). Indeed, caffeine use has been observed to increase neuromuscular conduction velocity and positively affect strength performance due to an increase in calcium in the sarcoplasmic reticulum in the muscle (8). A review of the literature has shown that moderate (9) or low (10) doses of caffeine significantly affect the physical and cognitive performance of players.

Carbohydrates are the primary source of energy for high-intensity activity. Consuming carbohydrates immediately before or after exercise has been shown to improve an athlete’s performance by increasing glycogen stores and delaying fatigue (11, 12). Studies have reported that the use of CHO during tennis matches maintains the blood glucose levels of the players; however, it does not affect the technical strokes of the players (13–15). Studies investigating the ergogenic effects of caffeine on tennis players have shown that low to moderate doses (3–6 mg/kg) of caffeine has an ergogenic effect on players’ groundstrokes (16–18). In some studies, CAF and CHO were used together. In these studies, CHO + CAF co-use was reported to increase energy availability (11), cognitive performance (19), intestinal glucose absorption, and exogenous CHO oxidation rate compared to CHO use alone (20, 21). Furthermore, CHO (6.4% carbohydrate) + CAF (4 mg/kg) supplementation improved badminton players’ serving accuracy and reaction time (22). An isotonic carbohydrate sports drink containing caffeine, consumed before and during a golf tournament, increased alertness in golf players and had a significant effect on the total putts made by the players (23). Kovacs et al. (24) showed that combining moderate doses of caffeine with a 7% CHO solution improved cyclists’ performance in time sessions more than using caf or CHO alone. This favorable effect is due to glucose absorption in the presence of CAF (25).

In the light of the literature review, only one study was found in the literature in which CHO and CAF were used together for tennis players. In the study conducted by Peilter et al. (26), tennis players played three tennis matches (each match lasted approximately 2 h) in 2 days. A standardized diet was administered to the players before each match. In addition, before, during, and after each match, players were given a sports drink (CHO + CAF) or PLA supplement. The study’s results demonstrated that players who received the sports drink performed longer rallies at higher heart rates (HR) during the 2-h matches and had lower Rating of Perceived Exertion (RPE) scores than those who received PLA alone. However, the authors did not investigate the effect of combined CHO + CAF supplementation on players’ groundstrokes in the study. One of the most critical parameters for players to succeed in a tennis match is their ability to sustain their strokes successfully. Therefore, the gap in the literature needs to be filled with more studies. In addition, the studies included CAF and PLA (18) or CHO and PLA (13) or CHO + CAF and PLA (26) supplements. However, these three ergogenic supplements have not been used together using the same protocol. Therefore, another rationale for conducting this study was to investigate how CHO, CAF, or CHO + CAF supplements, both separately and in combination, would affect the performance of tennis players.

Based on the above, this study aims to examine the effect of taking CAF_GUM_, CHO_GEL_, and CHO_GEL_ + CAF_GUM_ on players’ forehand and backhand strokes, countermovement jump (CMJ), HR, and RPE scores during a 2-h tennis training session. Above all, this study is the first to use CAF_GUM_, CHO_GEL_, and CHO_GEL_ + CAF_GUM_ supplements together, and it aimed to investigate which of these three ergogenic supplements would better affect tennis players. It was hypothesized that CHO_GEL_ + CAF_GUM_ during a training session could produce more beneficial effects on players’ groundstrokes and CMJ, HR, and RPE values than CHO_GEL_ and CAF_GUM_ supplements.

## 2 Materials and methods

### 2.1 Participants

**Figure 1:**
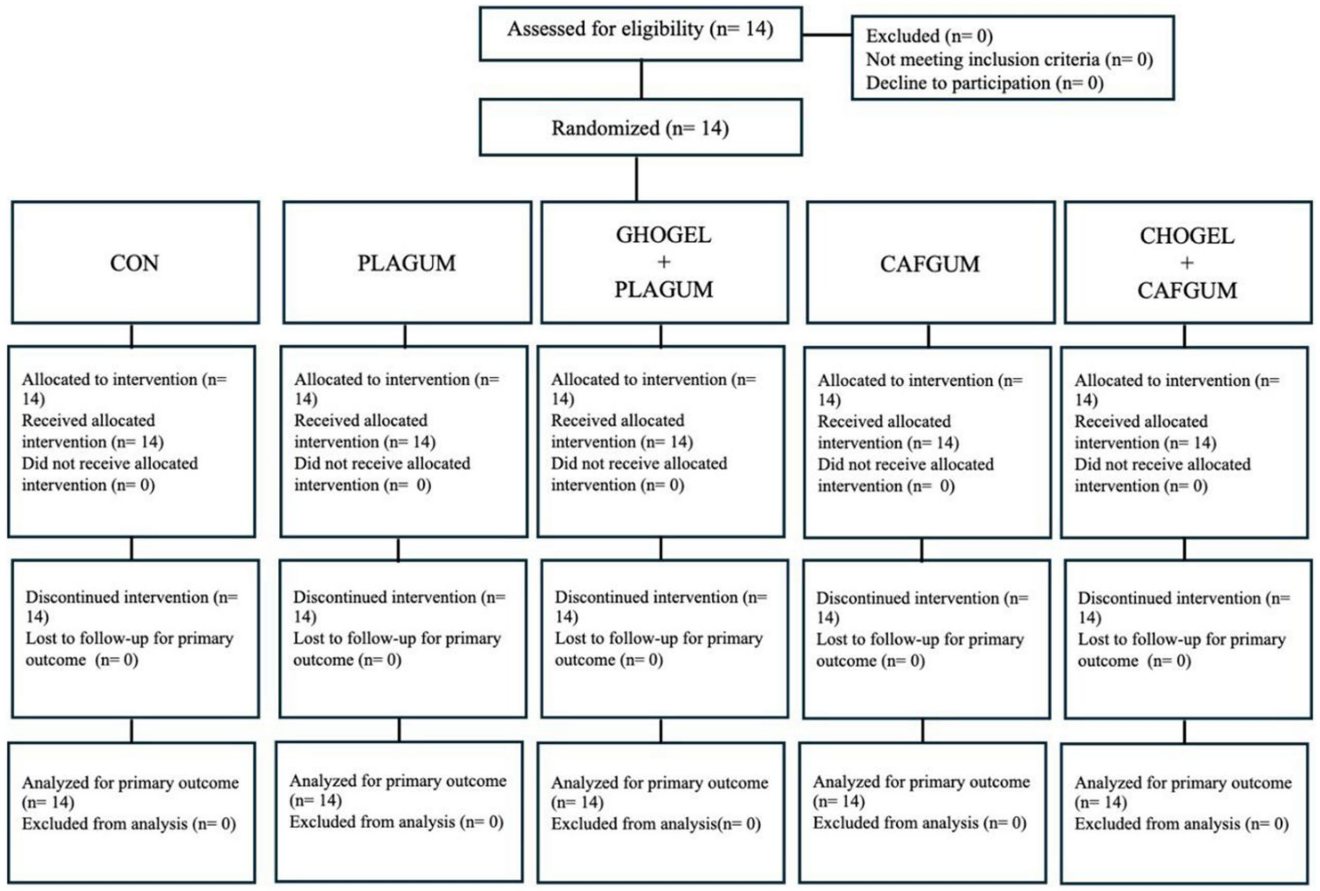
CONSORT 2025 flow diagram; CON, Control group; PLA_GUM_, Placebo gum; CHO_GEL_ + PLA_GUM_, Carbohydrate gel + Placebo gum; CAF_GUM_, caffeinated gum; CHO_GEL_ + CAF_GUM_, Carbohydrate gel + caffeinated gum.

**Figure 2:**
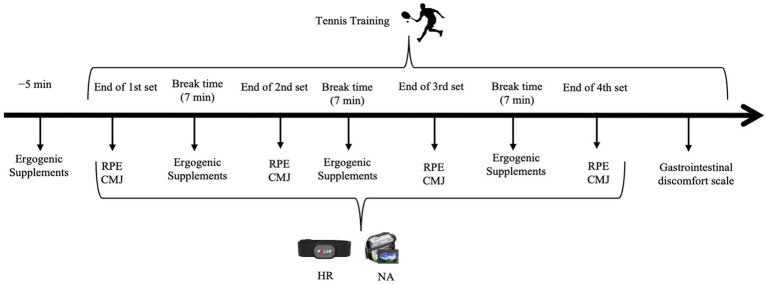
A schematic illustration of the experimental design. CMJ, Countermovement jumps; RPE, Ratings of perceived exertion; HR, heart rates; NA, Notational Analysis.

In this study, assuming a significance level of 0.05, a power of 0.80, and a correlation of 0.70 among five repeated measurements in each condition, a sample size of at least 10 participants was calculated to detect medium effect sizes. In determining the sample size, the research results of Gomes et al. were utilized, which investigated the technical and physical parameters of players using ergogenic supplements in a tennis match. To prevent a possible loss, we increased the required sample size to 14. The researchers sent personal invitations to tennis clubs and invited players to participate in the study. The study included 14 well-trained junior male tennis players (age: 15.93 ± 0.83 years, height: 173.86 ± 6.89 m, body mass: 60.64 ± 2.58 kg) between the ages of 15 and 17 years who regularly participated in tournaments (Tennis Europe and International Tennis Federation juniors). All participants were right-handed players who used primarily forehand strokes. All participants were chosen from convenience sampling, and there was no dropout. After the risks and benefits of the study were explained to each player, written informed consent was obtained from each participant and the participant’s parents (Figures 1, 2).

### 2.2 Experimental design

In this single-blind, randomized, placebo-controlled and crossover study, tennis players participated in five test days. Criteria for inclusion in the study included practicing tennis 4–6 days a week and attending tournaments regularly. The exclusion criteria included players who had a recent sports injury or who were not training regularly and not participating in the tournament. Five experimental sessions were performed during a tennis training session, the time interval between sessions was at least 72 h. To avoid changes in the players’ circadian rhythms, all tests were performed simultaneously (09:00 am, average temperature 25.5 ± 2.1° C, humidity 56.8 ± 9.2%) and on the same tennis court. Initially, the players’ demographic information and caffeine consumption frequency (75.21 ± 11.23 mg, range 61–86 mg per day) were determined, and anthropometric measurements were made (27). Additionally, a familiarization training session was given to each player. All players were given a list of caffeinated drinks and products and asked to avoid caffeine for at least 24 h before testing. Tennis players were instructed to consume the similar meal composition (25% fat, 15% protein, and 65% carbohydrate) before each testing session and to avoid high-intensity training sessions.

### 2.3 Training protocol

**Figure 3:**
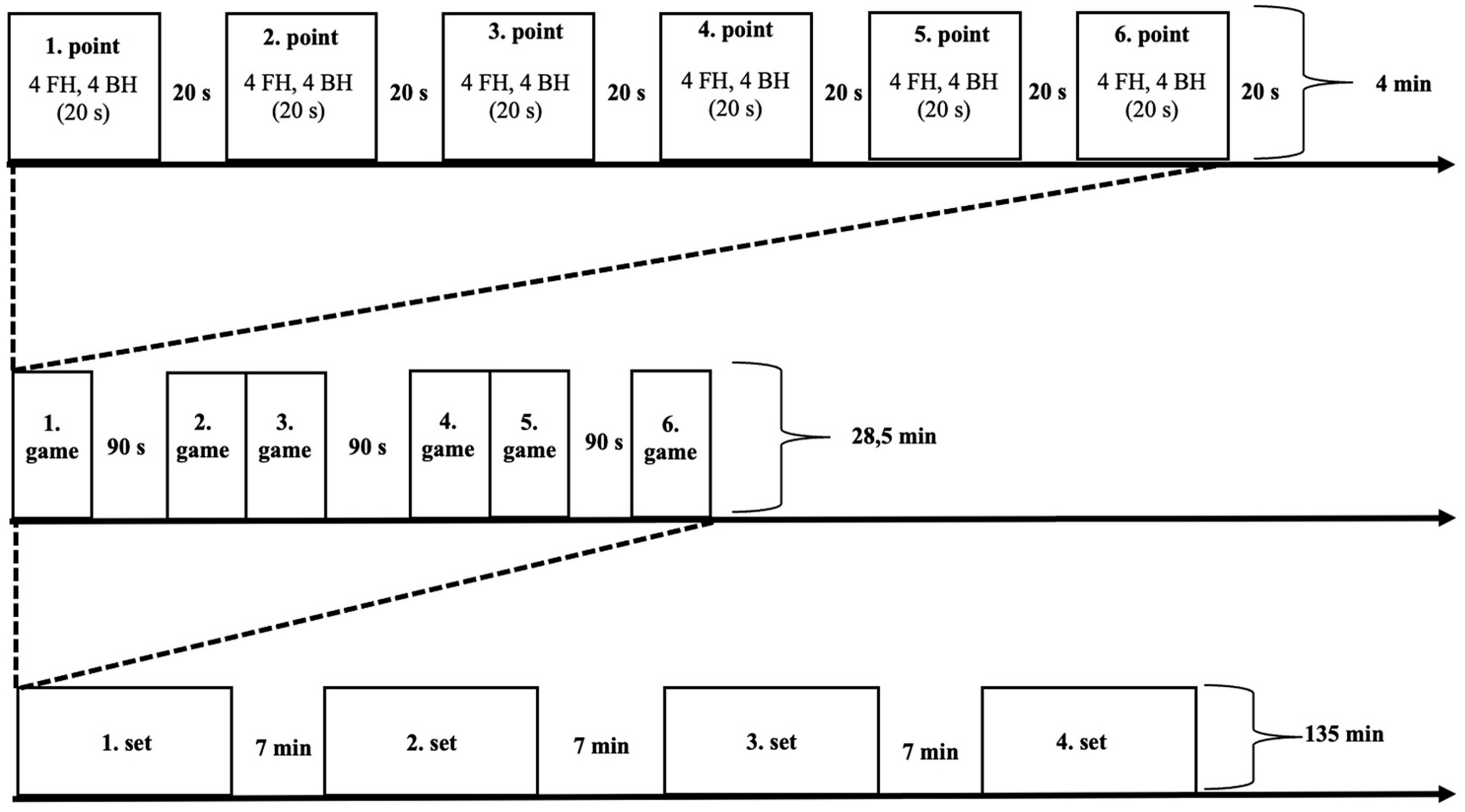
Training protocol; FH, Forehand; BH, Backhand.

**Figure 4:**
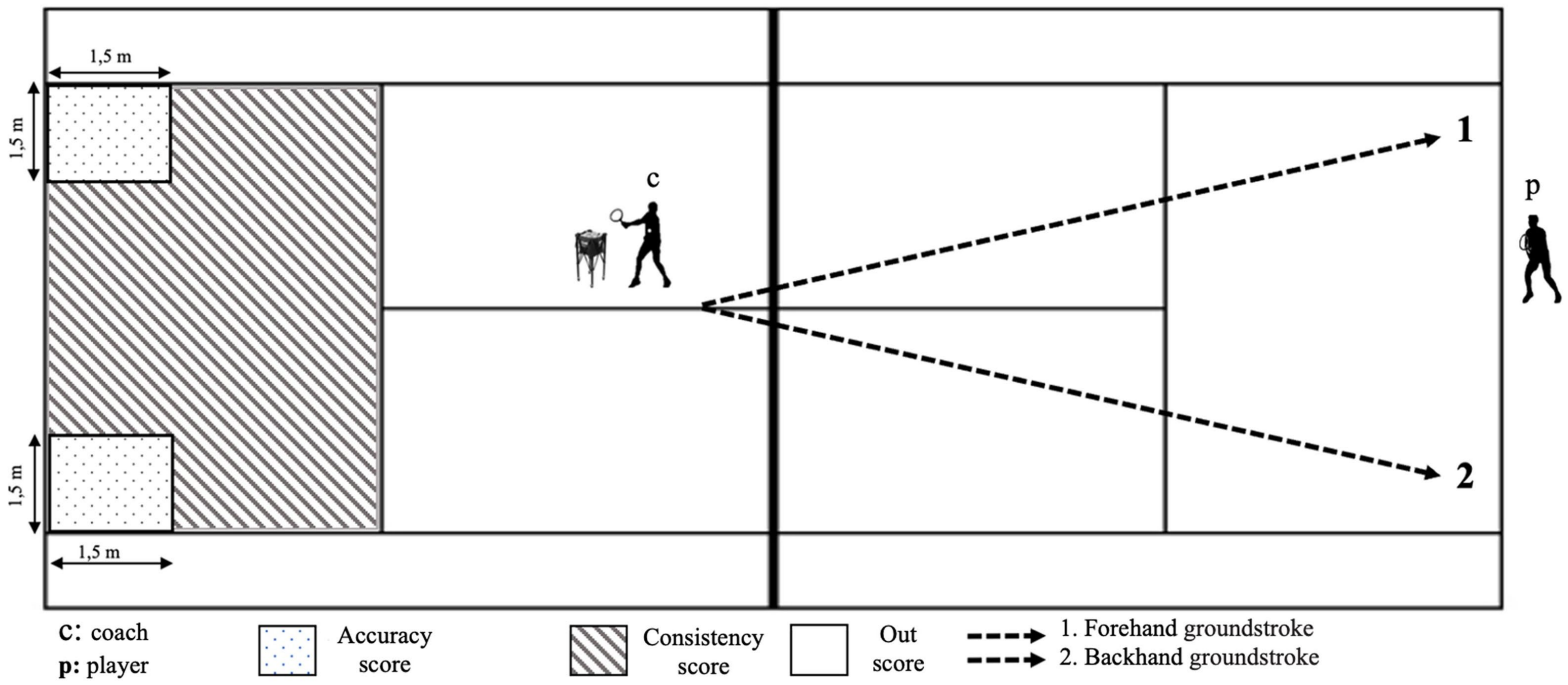
Court set-up and targeted area for the groundstrokes.

The training protocol was designed in accordance with the format of tennis matches. Each training session was designed as four sets (each set 28.5 min) and lasted 135 min (Figure 3). Players started dynamic warm-up for the training after taking a predetermined ergogenic supplement (28). Then, the players performed tennis-specific warm-up exercises on the court for 5 min (1 min from the mini-court, 4 min from the court baseline), followed by the training protocol. An experienced coach (certified level 3 by the Turkish Tennis Federation), who was not part of the research team, fed the ball first to the forehand side and then to the backhand side with the racket to the player waiting at the baseline (Figure 4). For each point, players performed eight groundstrokes (four forehands, four backhands, in approximately 20 s). The players were asked to make strokes with a quality similar to the strokes they make in matches (with high intensities). Players did their groundstrokes to their chosen area (cross-court or down the line). In order to determine a standardized intensity range during the training, the coach fed one ball to the players approximately every 2.5 s. All groundstrokes were recorded with a video camera. After each point the players rested passively for 20 s. At the end of each set (until the fourth set), the players received the same ergogenic supplement as before the training.

### 2.4 Notational analysis

The players’ ground strokes were evaluated during the training session to determine their performance. The target areas chosen for this evaluation were close to the baseline on the court, as suggested by a preliminary study by Vergauwen et al. (29). The study found that success rates increased when strokes were closer to the baseline and the opponent’s unsuccessful returns were closer to the sideline. To record the ground strokes made by the players, a digital camera (DSR-PDX10P, Sony, Japan) was used for evaluation. Before the training, the camera was mounted 5 m back from the court’s baseline and at 5–6 m height to see the entire court (30). For notational analysis, groundstrokes (forehand-backhand), successful strokes on the target, and unforced errors were evaluated from the obtained camera recordings (14, 29, 31). Another researcher with expertise in tennis counted strokes to reduce bias in the calculation of ground strokes in training. Only groundstroke valid scores (2 points) and consistency scores (1 point) were evaluated. Groundstroke valid and consistency scores were summed and converted to percentages for each player. Mean percentage scores were calculated for groundstroke valid and consistency total scores. Statistical analyses used raw percentage scores (4).

### 2.5 Determination of rating of perceived exertion

The Borg scale (6–20 points) was used to determine the intensity level of training sessions (32). The scale was administered to the players immediately after each set to determine the difficulty level of the set (13). Before the research, the details about the Borg scale application were explained to participants to ensure familiarization to the scale.

### 2.6 Determination of heart rate

Heart rates were recorded with a Polar H9 heart rate monitor (Polar Electro, Kempele, Finland) and the Polar Beat application on a smartphone (iPhone 11, Apple Park Way in Cupertino, California, United States). In accordance with the manufacturer’s instructions, the Polar H9 heart rate monitor was placed on the players’ chest over the heart. HR is updated every second, thanks to the small electrodes inside the heart rate monitor detecting the electrical stimuli of the heart (33). In this way, the maximal heart rates of the players were evaluated for each set in the training.

### 2.7 Countermovement jump

The CMJ test was performed between sets to determine the players’ neuromuscular fatigue levels. At the end of each set, the players’ heart rates were measured at approximately the first intensity zone of the Polar Team app, while the CMJ test was measured with the Myjump2 smartphone app. At the end of each set, the CMJ test was performed 2 times, and the best score was recorded. Hands remained fixed on the hips and the legs were required to remain fully extended during the flight phase of the jump. Upon stepping onto the ground participants were required to stand motionless for 2 s so that bodyweight could be accurately determined. The iPhone camera was focused on the feet only. The camera placement was adjusted to capture the participants’ full frame throughout the ground contact phase of the jump. The validity of this application has been examined in some studies (34, 35).

### 2.8 Gastrointestinal symptom rating scale

Revicki et al. (36) developed the Gastrointestinal Symptom Rating Scale (GSRS), a tool designed to assess the symptoms that often accompany discomfort in the gastrointestinal system. This scale, consisting of 15 items, employs a 7-point Likert-type scale to rate the severity of discomfort from “no discomfort” to “very severe discomfort.” The 15 items of the GSRS were categorized into five subscales through factor analysis: abdominal pain, reflux, diarrhea, indigestion, and constipation. Higher scores on the scale indicate more severe symptoms to evaluate the symptoms that frequently accompany gastrointestinal system discomfort.

### 2.9 Ergogenic supplements

Participants were randomly divided into five sessions CHO_GEL_ (21.1 g) + CAF_GUM_ (100 mg), CHO_GEL_ (21.1 g) + PLA_GUM_, CAF_GUM_ (100 mg), PLA_GUM_, and Control (CON) by the research team using Excel program. A recent study reported that PLA supplementation had a small to moderate positive effect on performance (37). Therefore, in this study, the CON session was also included to minimize this effect (37). It was recommended to take 30–60 g/h CHO during exercise (38). This study had a single-blind design. The research team covered all supplements given to the participants with black tape, so the participants did not know which supplement they were taking before and during the training. Supplements were given to the players 5 min before the training and before the 2nd, third, and fourth sets throughout the training. It has been reported in the literature that consuming 60–90 grams of CHO per hour may cause some stomach or intestinal problems in the body (38). Therefore, instead of taking a large amount of CHO (60–90 g/h) at a time, taking smaller amounts of CHO at certain intervals is recommended (e.g., when switching sides on the court). Additionally, players were only allowed to drink water during training, excluding supplements. A total of 84.4 g (1.4 g·kg^−1^) CHO_GEL_ and 400 mg (6.6 mg·kg^−1^) CAF_GUM_ were used in the training.

### 2.10 Data analysis

The SPSS (version 20) statistical program was used for data analysis. From descriptive statistics, categorical variables were presented as frequency and percentage, and continuous variables were presented as arithmetic mean and standard deviation. The Shapiro–Wilk test was used for the normality analysis of the data, and it was seen that the data was normally distributed. Then, one-way and two-way analyses of variance in repeated measures (ANOVA in repeated measures) were used. In technical, physiological, and physical data, “supplement × supplement, supplement × time” interactions, groundstrokes, HR, CMJ, and RPE scores were analyzed. The validity of the assumption of sphericity was determined by the Mauchly Test, and in cases where the assumption was not fulfilled, the Greenhouse–Geisser correction (>0.75) was used. When a significant main effect or interaction was detected, the Bonferroni post-hoc analysis was performed. Partial eta squared (ES) was calculated for the effect size of significant parameters. An effect size (ES) of <0.10 was classified as insignificant, 0.25–0.39 as moderate, and ≥0.40 as large (39). The significance level for all statistical analyses was set at (*p* = 0.05).

## 3 Results

### 3.1 Notational analysis

**Figure 5:**
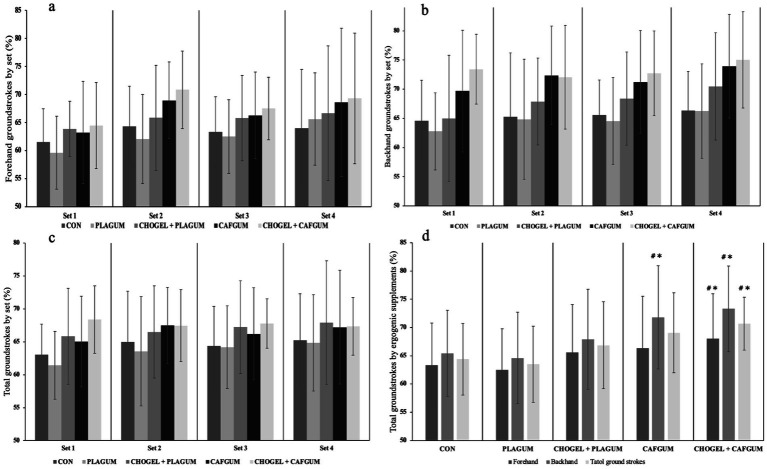
Groundstrokes during the training. **(a)** Forehand groundstrokes by set, **(b)** backhand groundstrokes by set, **(c)** total groundstrokes by set, and **(d)** total groundstrokes by ergogenic supplements. Groundstrokes during the training. CON, Control session; PLA_GUM_, Placebo gum; CHO_GEL_ + PLA_GUM_, Carbohydrate gel + Placebo gum; CAF_GUM_, caffeinated gum; CHO_GEL_ + CAF_GUM_, Carbohydrate gel + caffeinated gum; Values are the mean ± SD; ∗*p* < 0.05, compared with the CON; #*p* < 0.05, compared with the placebos.

The analyses revealed significant results among supplements for forehand strokes (*p* = 0.015; ES = 0.30) (Figure 5d). The CHO_GEL_ + CAF_GUM_ session made more successful strikes than the CON session by 4.72% (*p* = 0.017; ES = 0.29) and the PLA_GUM_ session by 5.57% (*p* = 0.035; ES = 0.34). Regarding the supplement x set interaction, the sessions had no significant difference (*p* = 0.312; ES = 0.86) (Figure 5a). Similarly, significant results were found among supplements for backhand strokes (*p* = 0.001; ES = 0.55). The CHO_GEL_ + CAF_GUM_ session made more successful strikes than the CON session by 7.86% (*p* = 0.001; ES = 0.45), the PLA_GUM_ session by 8.70% (*p* = 0.001; ES = 0.48), and the CHO_GEL_ session by 5.37% (*p* = 0.049; ES = 0.38), while the CAF_GUM_ session made more successful strikes than the PLA_GUM_ session by 6.350% (*p* = 0.038; ES = 0.85) and the CON session by 7.191% (*p* = 0.008; ES = 0.83) (Figure 5d). Regarding the supplement x set interaction, there was no significant difference among the sessions for backhand strokes (*p* = 0.518; ES = 0.58) (Figure 5b).

On the other hand, a significant difference was found among the supplements in the total forehand and backhand strokes performed in training (*p* = 0.001; ES = 0.52). The CHO_GEL_ + CAF_GUM_ session made more successful strikes than the CON session by 6.24% (*p* = 0.001; ES = 0.28) and the PLA_GUM_ session by 7.13% (*p* = 0.001; ES = 0.34). Also, the CAF_GUM_ session made more successful strikes than the PLA_GUM_ session by 3.30% (*p* = 0.009; ES = 0.15) (Figure 5d). Regarding the supplement x set interaction, there was no significant difference among the sessions in total forehand and backhand strokes (*p* = 0.408; ES = 0.71) (Figure 5c). Moreover, regarding the balls served by the coach (forehand and backhand strikes), no significant difference (*p* = 0.131) was found among CON (2.33 ± 0.20), PLA_GUM_ (2.32 ± 0.26), CHO_GEL_ + PLA_GUM_ (2.36 ± 0.15), CAF_GUM_ (2.39 ± 0.28), and CHO_GEL_ + CAF_GUM_ (2.34 ± 0.23) sessions (*p* = 0.131).

### 3.2 Rate of perceived exertion

**Figure 6:**
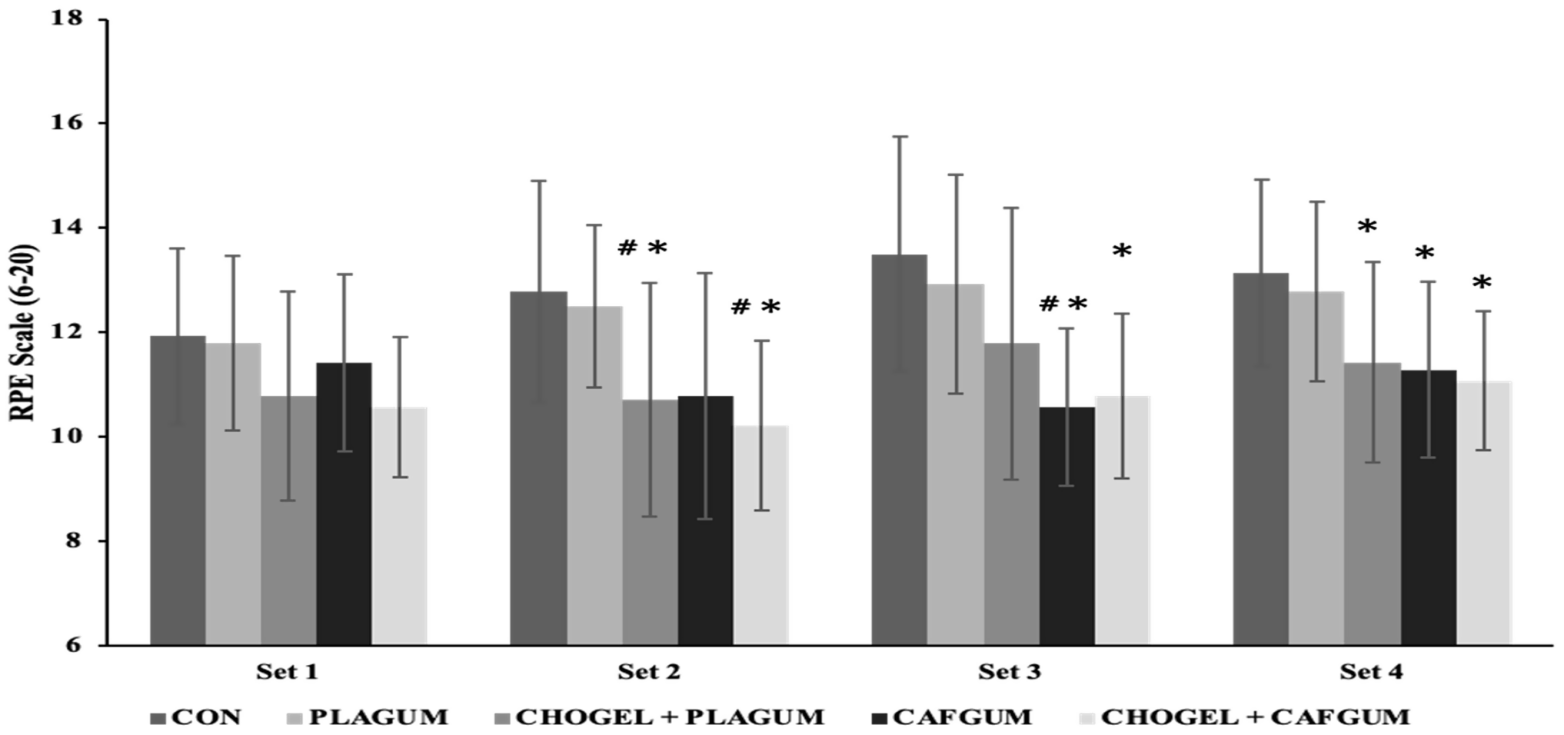
Rate of perceived exertion (RPE) at the end of each set during tennis training. rate of perceived exertion (RPE) at the end of each set during tennis training. CON, Control session; PLA_GUM_, Placebo gum; CHO_GEL_ + PLA_GUM_, Carbohydrate gel + Placebo gum; CAF_GUM_, caffeinated gum; CHO_GEL_ + CAF_GUM_, Carbohydrate gel + caffeinated gum; Values are the mean ± SD. ∗*p* < 0.05, compared with the CON; #*p* < 0.05, compared with the placebos.

A significant difference was found among the sessions’ RPE scores obtained during training (*p* = 0.001; ES = 0.43) (Figure 6). Similarly, a significant difference was detected between sets (*p* = 0.011; ES = 0.34). Also, regarding the supplement × time interaction, there was a significant difference among the sessions (*p* = 0.014; ES = 0.21). According to the results of Bonferroni’s post-hoc analysis, regarding total RPE values in training, the CHO_GEL_ + CAF_GUM_ session differed significantly from both the CON (*p* = 0.010; ES = −0.93) and the PLA_GUM_ sessions (*p* = 0.044; ES = −0.91), the CHO_GEL_ + PLA_GUM_ session differed significantly from both the CON (*p* = 0.005; ES = −0.85) and the PLA_GUM_ (*p* = 0.005; ES = −0.85), and the CAF_GUM_ session differed significantly from the CON session (*p* = 0.013; ES = −0.87). Regarding the set x set interaction, a significant difference was observed in time (*p* = 0.14; ES = 0.28). According to Bonferroni’s post-hoc analysis of the duration of the sets, the fourth set differed significantly from the 1st (*p* = 0.005; ES = −0.67) and the 2nd (*p* = 0.005; ES = −0.58) sets.

Regarding the supplement × time interaction, in the second set, the CHO_GEL_ + PLA_GUM_ session differed significantly from both the CON (*p* = 0.011; ES = −0.43) and the PLA_GUM_ (*p* = 0.027; ES = −0.42) sessions, while the CHO_GEL_ + CAF_GUM_ session differed significantly from both the PLA_GUM_ (*p* = 0.027, ES = −0.62) and the CON (*p* = 0.026; ES = −0.50) sessions. In the third set, the CAF_GUM_ session differed significantly from both the CON (*p* = 0.001; ES = −0.60) and the PLA_GUM_ (*p* = 0.003; ES = −0.54) sessions, while the CHO_GEL_ + CAF_GUM_ differed significantly from the CON session (*p* = 0.017; ES = −0.57). In the fourth set, the CHO_GEL_ + CAF_GUM_ session differed significantly from both the PLA_GUM_ (*p* = 0.003; ES = −0.42) and the CON (*p* = 0.002; ES = −0.35) sessions, the CAF_GUM_ differed significantly from the CON session (*p* = 0.016; ES = −0.47), and the CHO_GEL_ + CAF_GUM_ session differed significantly from the CON session (*p* = 0.020; ES = −0.57).

### 3.3 Countermovement jump

**Figure 7:**
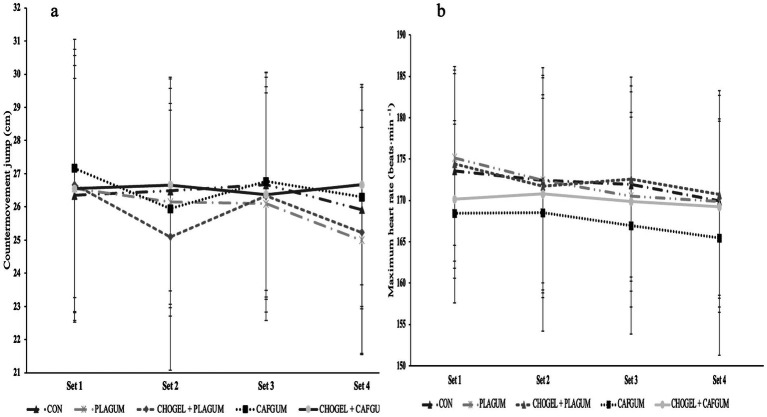
Countermovement jump and maximal heart rate at the end of each set during tennis training; CMJ and maximal heart rate at the end of each set during tennis training; **(a)** Countermovement jump, and **(b)** maximum heart rate at the end of each set during tennis training. CON, Control session; PLA_GUM_, Placebo gum; CHO_GEL_ + PLA_GUM_, Carbohydrate gel + Placebo gum; CAF_GUM_, caffeinated gum; CHO_GEL_ + CAF_GUM_, Carbohydrate gel + caffeinated gum; Values are the mean ± SD.

During the training, the CMJ values of the players were measured at the end of each set. Descriptive statistics of these values are presented in Figure 7a. According to the results, there was no significant difference between any of the supplements (*p* = 0.349; ES = 0.07). In the analysis of countermovement jump by time, no difference was found between supplements according to sets (*p* = 0.078; ES = 0.19). Supplement × time interactions were also insignificant (*p* = 0.139; ES = 0.12).

### 3.4 Maximal heart rate

It was observed that there was no significant difference between any of the supplements during the training (*p* = 0.0188; ES = 0.15). There was also no difference in HR values (*p* = 0.055; ES = 0.21) of the supplement sessions between sets (Figure 7b). There is no significant difference between the interaction of supplement and time (*p* = 0.250; ES = 0.09).

### 3.5 Gastrointestinal symptom rating scale

According to the results of the GSRS, which was applied to determine the gastrointestinal discomfort that the supplementation may cause, there was no significant difference between any supplement sessions in symptoms such as reflux syndrome, abdominal pain, constipation syndrome, diarrhea syndrome, and indigestion syndrome (*p* = 0.356). CON, PLA_GUM_, and CAF_GUM_ sessions reported the lowest scores among the variables. Although not statistically significant, CHO_GEL_ + PLA_GUM_ and CHO_GEL_ + CAF_GUM_ sessions scored higher on the abdominal pain variables than the other groups (Table 1).

**Table 1:** Gastrointestinal symptom rating scale.

Variables	Control	PLAGUM	CHOGEL+PLAGUM	CAFGUM	CHOGEL+CAFGUM
Abdominal pain	1	1	1.71	1	1.64
Reflux syndrome	1	1	1	1	1
Diarrhea syndrome	1	1	1	1	1
Indigestion syndrome	1	1	1	1	1
Constipation syndrome	1	1	1	1	1

CON, Control group; PLA_GUM_, Placebo gum; CHO_GEL_ + PLA_GUM_, Carbohydrate gel + Placebo gum; CAF_GUM_, caffeinated gum; CHO_GEL_ + CAF_GUM_, Carbohydrate gel + caffeinated gum; Values are the mean.

## 4 Discussion

In this study, CHO_GEL_, CAF_GUM_, and CHO_GEL_ + CAF_GUM_ supplements were used together for the first time to determine the effectiveness of ergogenic supplements on tennis players’ physical and technical performance. This study analyzed the effects of ergogenic supplements used before and during training on parameters such as forehand, backhand, HR, CMJ, and RPE of tennis players. Although our study findings showed that CHO_GEL_, CAF_GUM_, and CHO_GEL_ + CAF_GUM_ supplements positively affected the performance of the players, there was no significant difference in the groundstrokes, RPE scores, HR, and CMJ test results of the players between the ergogenic supplements used in the study. However, among all supplements, CHO_GEL_ + CAF_GUM_ supplementation was found to have the highest ergogenic effect on players’ forehand, backhand (Figure 5d), and RPE scores in both PLA_GUM_ and CON sessions (Figure 6). However, all ergogenic supplements did not positively affect the CMJ test and maximum heart rate. These results partially support the hypothesis of our study.

CHO_GEL_ and CAF_GUM_ used alone did not significantly affect the players’ forehand strokes during the 2-h, 15-min training period compared to the PLA_GUM_ and CON sessions. However, CHO_GEL_ + CAF_GUM_ supplementation showed a more significant difference in the forehand strokes of the players than the other supplementation sessions. This finding is important because in tennis matches, forehand shots are generally made more often than backhand shots (40). Therefore, more successful forehand strokes may increase players’ chances of winning a match. The time between rallies during the training was approximately 2.5 s, which is in line with the current protocol (26). However, although the time between rallies remained constant throughout the match, the CHO_GEL_ + CAF_GUM_ session made the fewest unforced errors. We can say that the biggest reason for this is the positive effect of the combined supplement used. Based on these results, CHO_GEL_ + CAF_GUM_ supplementation, which will be used periodically in training, will help players maintain their groundstroke performance and have fewer unforced errors (22, 23).

In prolonged matches, the unforced error rate in ground strokes may increase toward the match’s final stages due to fatigue (4). In studies in the literature, it was reported that players’ groundstroke performance decreased due to fatigue, but low (14) and moderate doses of caffeine (16–18) increased the performance of players. CHO, another ergogenic supplement, also positively affects tennis players’ ground strokes (17). However, according to the literature, it is not known how the use of CHO_GEL_ + CAF_GUM_ will affect ground strokes, and only one study showed that players using CHO + CAF performed longer rallies at higher heart rates. However, according to the literature, it is not known how the use of CHO_GEL_ + CAF_GUM_ will affect groundstrokes, and only one study showed that players using CHO + CAF performed longer rallies at higher heart rates (26). However, this study did not report any findings regarding the effect of combined supplement use on players’ groundstroke performance. To elucidate this, in the present study, CHO_GEL_ + CAF_GUM_ supplementation improved the groundstroke performance of the players more than CON (6.24%) and PLA_GUM_ (7.13%) sessions during a 2-h 15-min tennis training session. In addition, although players made more successful shots during CHO_GEL_ + CAF_GUM_ session than CAF_GUM_ and CHO_GEL_ + PLA_GUM_ sessions, there was no significant difference between the sessions. In a study conducted on badminton players, players taking CHO_GEL_ + CAF_GUM_ supplements were found to have more successful serves than players taking CAF_GUM_ or CHO_GEL_ supplements alone. These results are partially parallel to the results of our study (22).

This research protocol did not allow the verification of the mechanisms underlying the favorable performance enhancement. However, the current performance enhancement may be related to caffeine-induced changes in the central nervous system. This is because caffeine stimulates the central nervous system and can block adenosine-specific receptors that increase the release of norepinephrine, dopamine, acetylcholine, and serotonin, among other neurotransmitters (41). In addition, some studies have reported that CHO + CAF supplementation increases muscle energy availability (11), cognitive performance (19), intestinal glucose absorption, and exogenous CHO oxidation rate (21). For these reasons, CHO_GEL_ + CAF_GUM_ supplementation is predicted to result in excellent performance enhancement in players. However, more studies are needed to confirm this fully.

Caffeine use has been shown to positively affect RPE scores during submaximal exercise (42, 43), and CHO use has been shown to positively affect RPE during endurance exercise (44). It has also been noted that the combined use of caffeine and carbohydrates produced significant differences in RPE scores (22, 26). This ergogenic factor is that caffeine easily crosses the blood–brain barrier thanks to its special chemical structure and acts on adenosine receptors (A1, A2), thus blocking pain-producing receptors in the body (45). In addition, using CHO preserves glycogen stores and thus contributes to maintaining blood glucose levels (44). In our study, tennis players using CHO_GEL_ + PLA_GUM_, CAF_GUM_, or CHO_GEL_ + CAF_GUM_ supplements reported less training difficulty compared to CON and PLA_GUM_ sessions. Based on this finding, ergogenic supplements affect the fatigue felt by players, although there is no difference in mean or maximum HRs when training with a standardized protocol. In fact, this finding is important because high-performance adolescent tennis players train twice a day. Therefore, these ergogenic supplements before training may delay the fatigue felt by the players during the next training session. Future studies must test parameters affecting tennis performance during or after training or matches to confirm this.

During a three-hour tennis match, it has been reported that the average HR of high-performance players gradually decreased as the effective playing time decreased (46). In the present study, neither maximum HR nor mean HR values changed in any supplement session during training lasting more than 2 h (Figure 7b). This may be due to the shorter training duration in this study and, most importantly, the frequency of ball feeding (~2.5 s), which remained constant. Peilter et al. (26) found higher HR values in players consuming a combined CHO + CAF drink than those consuming PLA alone. However, the authors noted that the higher mean HR in players consuming CHO + CAF compared to the CON session may be related to increased intensity rather than a direct effect of CAF on the cardiovascular system. In fact, this argument is consistent with the literature because neither the use of CAF_GUM_ (14) nor CHO_GEL_ (15) showed a significant difference in the HR values of tennis players. In addition, a meta-analysis showed that CAF used at a moderate dose (6 mg/kg) did not alter players’ HR values during exercise (42). In the current study, CMJ tests performed after each training session to determine players’ neuromuscular fatigue did not reveal any difference between any supplements. In line with our study results, in one study, the CHO and PLA_GUM_ sessions did not differ in terms of CMJ test results measured after a 3-h match (16). In another study, Filip-Stachnik et al. (47) found no significant difference in the jumping performance of female volleyball players using CAF_GUM_ (400 mg).

Although CHO used in different forms (gel and liquid sources) had the same ergogenic effect on players, gastrointestinal (GI) discomfort was observed in some players after CHO_GEL_ use (48). In a study, CHO_GEL_ used during running exercise (70% VO_2_max) was observed to cause some problems in the GI wall of players at the end of exercise (49). Ingestion of concentrated CHO gels, such as gel products, can lead to GI disturbances (such as malabsorption and gastric distension) as a result of the osmotic diversion of water into the intestine (50). A study involving tennis players reported that using 7.5 g carbohydrate/100 mL may cause some GI disturbance (51). These results are inconsistent with our study results. In our study, players consumed CHO_GEL_ (21 g) or CHO_GEL_ (21 g) + CAF_GUM_ (100 mg) after each training set (25 min apart). No GI disturbances were observed in the players due to using 84 g CHO_GEL_ and 400 mg CAF_GUM_ during more than 2 h of training. Kovacs (38) recommended that tennis players take small amounts of CHO_GEL_ at regular intervals instead of large amounts before training or matches. In line with these recommendations, fructose-free CHO_GEL_ was used in low amounts and at regular intervals in our study protocol, which may have prevented the occurrence of GI disturbance symptoms in the participants.

The limitation of this study is that the players’ blood glucose values were not obtained. Therefore, which supplements affected the players’ blood sugar values during and after training is unclear. Future studies can address this issue by taking blood glucose measurements. Meanwhile, only 14 male and adolescent tennis players participated in the study. Therefore, researchers need to consider this in the evaluations to be made. Since Global Positioning System (GPS) equipment was not used in our study, parameters such as acceleration-deceleration and total distance covered by the players during training were not controlled. Controlling these parameters in future studies will contribute to the literature. Future studies should be double-blind, and a ball machine should be used instead of a trainer to feed the ball. Our research was similar to the match simulation in training; however, it is necessary to investigate the effect of these supplements in competitive conditions and the players’ hydration values at the end of the match.

Although all the supplements used in the study (CHO_GEL_, CAF_GUM_, and CHO_GEL_ + CAF_GUM_) produced some ergogenic effects, the combined supplementation provided the most significant effect. Therefore, taking CHO_GEL_ (21.1 g) + CAF_GUM_ (100 mg) before and during intense training sessions (one CHO_GEL_ and one piece of CAF_GUM_ every 25 min) may have a positive effect on players’ groundstrokes and RPE scores. Especially during intense training times (double training sessions per day), this supplement will help players improve their performance in the next training session. Furthermore, the developed ergogenic supplementation protocol is an effective tool to measure the effect of fatigue and experimental strategies on tennis skills. However, it is also recognized that training cannot have a true competitive essence.

## 5 Conclusion

Junior tennis players demonstrated significantly better values in tennis training parameters after using CHO_GEL_ + CAF_GUM_ supplementation before and during training compared to PLA ant CON. CHO_GEL_ + CAF_GUM_ supplementation had the best ergogenic effect on players’ groundstroke and RPE scores compared to CON and PLA_GUM_ sessions. This could lead to a cumulative increase in players’ performance throughout the tennis season. Future studies could investigate how these supplements affect players’ technical and physiological state under match conditions.

## Data Availability

The raw data supporting the conclusions of this article will be made available by the authors, without undue reservation.
